# Multi-Parameter Analysis of Nanoplastics in Flow: Taking Advantage of High Sensitivity and Time Resolution Enabled by Stimulated Raman Scattering

**DOI:** 10.1021/acs.analchem.3c05881

**Published:** 2024-05-21

**Authors:** Maximilian J. Huber, Liron Zada, Natalia P. Ivleva, Freek Ariese

**Affiliations:** † Chair of Analytical Chemistry and Water Chemistry, Institute of Water Chemistry, Technical University of Munich, Lichtenbergstr. 4, 85748 Garching, Germany; ‡ LaserLaB Amsterdam, Department of Physics and Astronomy, Vrije Universiteit Amsterdam, 1081 HV Amsterdam, The Netherlands

## Abstract

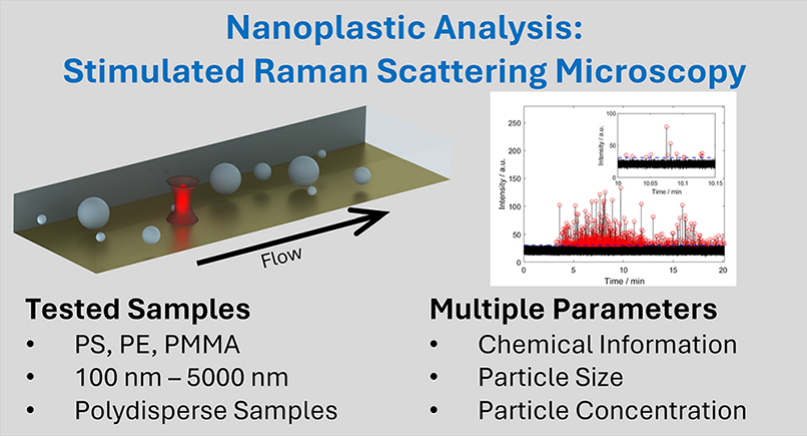

Here, we demonstrate the detection of nanoplastics (NPLs) in flow with stimulated Raman scattering (SRS) for the first time. NPLs (plastic particles <1000 nm) have recently been detected in different environmental samples and personal care products. However, their characterization is still an analytical challenge. Multiple parameters, including size, chemical composition, and concentration (particle number and mass), need to be determined. In an earlier paper, online field flow fractionation (FFF)-Raman analysis with optical trapping was shown to be a promising tool for the detection of particles in this size range. SRS, which is based on the enhancement of a vibrational transition by the matching energy difference of two laser beams, would allow for much more sensitive detection and, hence, much shorter acquisition times compared to spontaneous Raman microspectroscopy (RM). Here, we show the applicability of SRS for the flow-based analysis of individual, untrapped NPLs. It was possible to detect polyethylene (PE), polystyrene (PS), and poly(methyl methacrylate) (PMMA) beads with diameters of 100–5000 nm. The high time resolution of 60.5 μs allows us to detect individual signals per particle and to correlate the number of detected particles to the injected mass concentration. Furthermore, due to the high time resolution, optically trapped beads could be distinguished from untrapped beads by their peak shapes. The SRS wavenumber settings add chemical selectivity to the measurement. Whereas optical trapping is necessary for the flow-based detection of particles by spontaneous RM, the current study demonstrates that SRS can detect particles in a flow without trapping. Additionally, the mean particle size could be estimated using the mean width (duration) and intensity of the SRS signals.

## Introduction

The recent detection of nanoplastics (NPLs, plastic particles <1000 nm^1−3^) in various locations, ranging from soil,^4^ snow,^5,6^ polar ice,^7^ to aquatic environments,^8−10^ supports the assumed widespread environmental contamination with these anthropogenic particulate pollutants. Because of their small size, large surface-to-volume ratio, and ability to cross biological membranes, their environmental fate and (eco)toxicological effects can be quite different from those of microplastics.^11^ Depending on their origin, they can be classified as primary or secondary NPLs. Secondary NPLs are generated from larger plastic particles via different degradation and fragmentation processes.^12^ It was suggested for microplastics that the number of particles increases exponentially for smaller sizes.^13^ The size distribution of nanoplastics is still unknown. Considering the vast amount of plastic debris in the environment, this is assumed to be the main source of NPLs.^14^ Intentionally manufactured NPLs are called primary NPLs. These can be released from consumer products, such as paints, adhesives, coatings,^15^ or personal care products^16^ during their life cycle. Whereas the addition of plastic particles as scrubbing agents in personal care products was banned in the United States, Canada, and the European Union, they are still used in other parts of the world.^16,17^ Furthermore, primary NPLs can also be released during applications and processes, including plastic manufacturing,^18^ 3D printing,^19^ and medical applications for drug delivery.^20^ Especially for these point sources of NPL contamination, emission reduction, and monitoring would be much simpler compared to the widespread contamination by secondary NPLs.

However, the reliable analysis of NPLs is still very challenging. For size characterization, several techniques, including dynamic light scattering (DLS), nanoparticle tracking analysis (NTA), multiangle light scattering (MALS), tunable resistive pulse sensing (TRPS), and centrifugal liquid sedimentation (CLS), have been tested, and their advantages and limitations have been evaluated to a certain degree.^21,22^ For most samples, however, even with a pure size determination, it would not be sufficient to distinguish NPLs from nonplastic particles in the same size range since determining the polymer type would be important for identifying sources. Thus, chemical information is crucial for the assessment of NPL contamination. To this end, several methods have been developed in the past, including mass spectrometric techniques in combination with fractionation techniques^23,24^ and vibrational spectroscopic methods coupled to scanning probe microscopy^25,26^ or field flow fractionation (FFF).^22,27^ In general, combined techniques can provide information on several sample properties, such as size, size distribution, and chemical composition.^3,22,28^ For example, online-FFF-Raman microspectroscopy (RM) is enabled by optical trapping (OT) and can detect and identify NPLs below the diffraction limit in flow using MALS for size determination.^22,27^ However, due to the low sensitivity of spontaneous RM, OT and signal collection over a relatively long time (several seconds) are necessary to acquire a spectrum of sufficient signal-to-noise ratio for NPL identification.^29^ This limits the type of particles with respect to their size, shape, and optical properties that can be analyzed with the FFF-RM setup. Furthermore, it is difficult to distinguish larger particles from a cluster of smaller ones. The application of OT in combination with RM was first demonstrated for NPLs by Gillibert et al.,^29^ and the detection of single particles (extracellular vesicles) was reported by Enciso-Martinez et al.^30^ using a stationary, not flow-based setup. Schwaferts et al. showed that the hyphenation of RM with FFF-MALS as a preceding fractionation and size characterization technique can be a powerful combination for the analysis of NPLs.^27^ The OT necessary for this online coupling describes the forces (traditionally called scattering and gradient forces) of a focused laser beam that act on particles in the micro- and nanometer range. For spherical particles, the scattering force points in the direction of light propagation and is caused by the momentum transfer of the photons to the particle, while the gradient forces point in the direction of the highest light intensity. This can be explained by the interaction of the inhomogeneous electric field (intensity gradient of the laser focus) and the induced dipoles in dielectric particles. Thus, particles are pulled toward the center of the focal spot slightly below the focal plane where the two forces are in equilibrium.^31^ Experiments can be performed in 2D and 3D mode. 2D describes the trapping of particles by pushing them against a surface, while 3D trapping is used to capture particles free in suspension.

In contrast to spontaneous RM, coherent Raman scattering (CRS) techniques, mainly coherent anti-Stokes Raman scattering (CARS) and stimulated Raman scattering (SRS), can provide much stronger signals but at the expense of spectral information.^32^ Thus, integration times can typically be reduced from seconds for spontaneous RM to microseconds for CRS techniques.^33,34^ Additionally, SRS benefits from the lack of nonresonant background, and when operated in the NIR range, it is rather immune to the strong fluorescence interference that often hampers spontaneous RM analysis of environmental and other types of complex samples.^35^ Current microplastic studies in our lab (Konings et al., manuscript in preparation) show that, especially in the case of pigmented polymers, fluorescence interference is a major obstacle in conventional RM analysis but not under SRS conditions. For a signal to be generated in SRS, the photon energy difference of two laser beams must match the vibrational state of the molecules that reside in the overlap of the focal volumes of the two focused beams. Most SRS setups use picosecond pulse trains as an optimal compromise of signal intensity and spectral bandwidth. With such SRS setups, only a small bandwidth of the Raman spectrum (typically a single vibrational band) can be observed simultaneously. This offers a certain degree of selectivity for distinguishing different polymer types, but there will be less spectral information than in the case of spontaneous RM. Since the SRS signal (in our setup stimulated Raman loss of the pump beam) is only a small fraction of the pump beam intensity, the Stokes beam is amplitude modulated. This modulation is transferred to the pump beam with the amplitude of the modulation transfer being proportional to the Raman cross section and the analyte concentration in the focal volume.^36^ Thus, in principle, SRS can be used for quantitative analysis. A lock-in amplifier (LIA) is used for the sensitive detection of the SRS signal. The LIA mixes the detected signal with a reference frequency used for the Stokes beam modulation and applies a low pass filter. The result is a DC signal proportional to the amplitude of the detected signal (pump beam) modulation, i.e., the SRS signal.^37^ Although SRS has been applied successfully for the analysis of microplastics on a filter,^34^ in a silicone tissue phantom,^38^ and in suspension,^39,40^ the detection of plastic particles below 1 μm (NPLs) with SRS has not been demonstrated so far. Since NPLs cannot be resolved with optical methods at the single particle level due to the diffraction limit, the analysis on a filter would not enable us to distinguish single particles from a cluster of smaller ones.

In this work, the applicability of SRS for the analysis of NPLs in flow-based systems is tested for the first time, while the coupling is achieved by a flow cell. A set of polyethylene (PE), poly(methyl methacrylate) (PMMA), and polystyrene (PS) particles in the size range of 100–5000 nm was chosen, all of which could be detected with the used setup. Due to the higher sensitivity and significantly reduced integration time compared to spontaneous Raman, SRS signals of individual particles could be obtained in this flow-based setup. The peak shape gives further insight into the degree of OT, and for untrapped particles, it provides an indication of the particle diameter. Furthermore, the detection of individual particles allows for a determination of the concentration (particle number) after calibration.

## Methods and Materials

### Sample Preparation

In this study, PE, PMMA, and PS nanoplastic samples of various sizes and size distributions were used to investigate the applicability of SRS for NPL analysis. An overview of the samples and their properties is given in Table 1. The stock solutions were diluted in a surfactant solution of 0.0125% NovaChem100 (Postnova Analytics GmbH (PN), Germany) in water (Milli-Q) to keep the particles in suspension. This solution was also used as a carrier liquid for in-flow detection. Due to the low sample stability of the samples PE*X*, PS100, PS230, and PS*X*, the suspensions were treated in a sonication bath (Branson 2150, Branson Ultrasonics Corp., USA) for 10 min before and after dilution.

**Table 1:** List of Samples and Mixtures Used in This Study

sample	supplier	stock concentration (% mass)	expected size (Shape)	description
PE*X*^a^	SINTEF Industry, Norway	5%	400 nm (spherical)	polyethylene (PE) particles in water stabilized with 0,5% Tween 80, polydisperse
PMMA100	microParticles GmbH, Germany	5%	100 nm (spherical)	in water, monodisperse
PMMA500	microParticles GmbH, Germany	5%	500 nm (spherical)	in water, monodisperse
PS100	BS-Partikel GmbH, Germany	5%	100 nm (spherical)	in water, monodisperse
PS230	BS-Partikel GmbH, Germany	5%	230 nm (spherical)	in water, monodisperse
PS300	Duke Standards, Thermo Fisher Scientific, USA	5%	300 nm (spherical)	in water, monodisperse
PS350	Duke Standards, Thermo Fisher Scientific, UAS	5%	350 nm (spherical)	in water, monodisperse
PS430	Duke Standards, Thermo Fisher Scientific, USA	5%	430 nm (spherical)	in water, monodisperse
PS600	Duke Standards, Thermo Fisher Scientific, USA	5%	600 nm (spherical)	in water, monodisperse
PS1000	Applied Microspheres, The Netherlands	1%	1000 nm (spherical)	in water, monodisperse
PS5000	BS-Partikel GmbH, Germany		5000 nm (irregular)	powder, monodisperse
PS*X*^a^	SINTEF Industry, Norway	16%	250 nm (spherical)	PS particles in water stabilized with 3 g/L sodium lignosulfonate (NaLS), polydisperse

a “*X*” denotes polydisperse samples of polystyrene (PS*X*) and polyethylene (PE*X*).

### Instrumental Setup

A manual injector (9725i, IDEX Health & Science, USA) with a sample loop of 19.6 μL was used in combination with a solvent degasser (PN7520, PN) and an isocratic pump (PN1130, PN) to introduce the sample into a Raman flow cell. The general construction of the flow cell (Figure S1 in the Supporting Information (SI)) was previously described by Schwaferts et al.^27^ and has a 350 μm spacer and two layers of 50 μm thick double-sided adhesive tape. This results in a channel height of 450 μm and a channel width of 1500 μm. Unlike earlier RM measurements, the foci were placed 30 μm above the flow cell base. This was due to the high background signal for both flow cell bases (gold-plated steel and polycarbonate (PC)), which might have been caused by thermal effects and/or the surfactant adsorbed to the channel surface. Whereas for SRS detection in epi-mode the same gold-plated steel base was used, for SRS detection in transmission mode, a similar PC base was manufactured.

The home-built SRS setup (see Figure S2 in the Supporting Information) is based on a ps-pulsed laser at 1064 nm (Stokes beam); its frequency-doubled output operates an Optical Parametric Oscillator (OPO) as the pump beam. The 1064 nm beam is modulated using an acousto-optic modulator (AOM). The difference in photon energy between the two beams is tuned to a specific vibration of the target polymer compound. The two beams are overlapped in time and space and are focused inside the flow cell using a Zeiss 7MP scanning microscope with a C-achroplan W 32× water immersion objective with a numerical aperture of 0.85 (Carl Zeiss Microscopy GmbH, Germany). The presence of the target polymer in the focal volume leads to a decrease in the pump intensity, which is detected as the SRS signal. A more detailed description of the setup) was provided previously.^34,35^

For comparison, also an *alpha300 apyron* Confocal Raman microscope (WITec GmbH, Germany) equipped with a 532 nm DPSS laser and a W-plan apochromat 63× water immersion objective with a numerical aperture of 1.00 (Carl Zeiss Microscopy GmbH, Germany) was used.

The flow rate was set to 0.1 mL min^–1^ (corresponding with a velocity of 2.5 mm s^–1^) for all measurements. Each SRS measurement was conducted over the whole injection time of 20 min with a 60.5 μs acquisition time. For the lock-in amplifier (LIA) settings, a time constant of 13 μs and a filter order of 8 were chosen to support this time resolution.^37^ For the detection in epi-mode, the laser power at the sample was set to 33 mW for the 1064 nm beam (Stokes) and 17 mW for the OPO output beam (pump), whereas for detection in transmission mode, the laser powers were set to 66 and 34 mW, respectively. The higher laser powers in transmission mode could be achieved due to the removal of a beam splitter, resulting in better light transmission onto the sample. The transmission geometry also means that a larger fraction of pump photons reach the detector. To detect NPLs, a suitable Raman band in the CH-stretch region was chosen to set the OPO output wavelength: (for PS: 803.4 nm = 3049 cm^–1^, for PE: 814.6 nm = 2877 cm^–1^, for PMMA: 809.5 nm = 2955 cm^–1^). The selected wavenumber for PS is in the aromatic C–H stretch region and therefore selective, when considering only these polymer types. The other wavenumbers were selected due to their high relative intensity. As they are in the alkyl C–H stretch region, they are not specific for a certain material, and for a more definitive chemical identification, additional measurements at different wavenumbers would have to be conducted. In fact, our current SRS setup is tunable over the 1100–3900 cm^–1^ range, and targeting polymer particles at more specific fingerprint vibrations was demonstrated for environmental microplastics by Zada et al.^34^ The acquired data were evaluated using a MATLAB script (Version R2022b for Windows, MathWorks) provided in the Supporting Information.

## Results and Discussion

### Detection of NPLs in Flow-Based Setups

**Figure 1:**
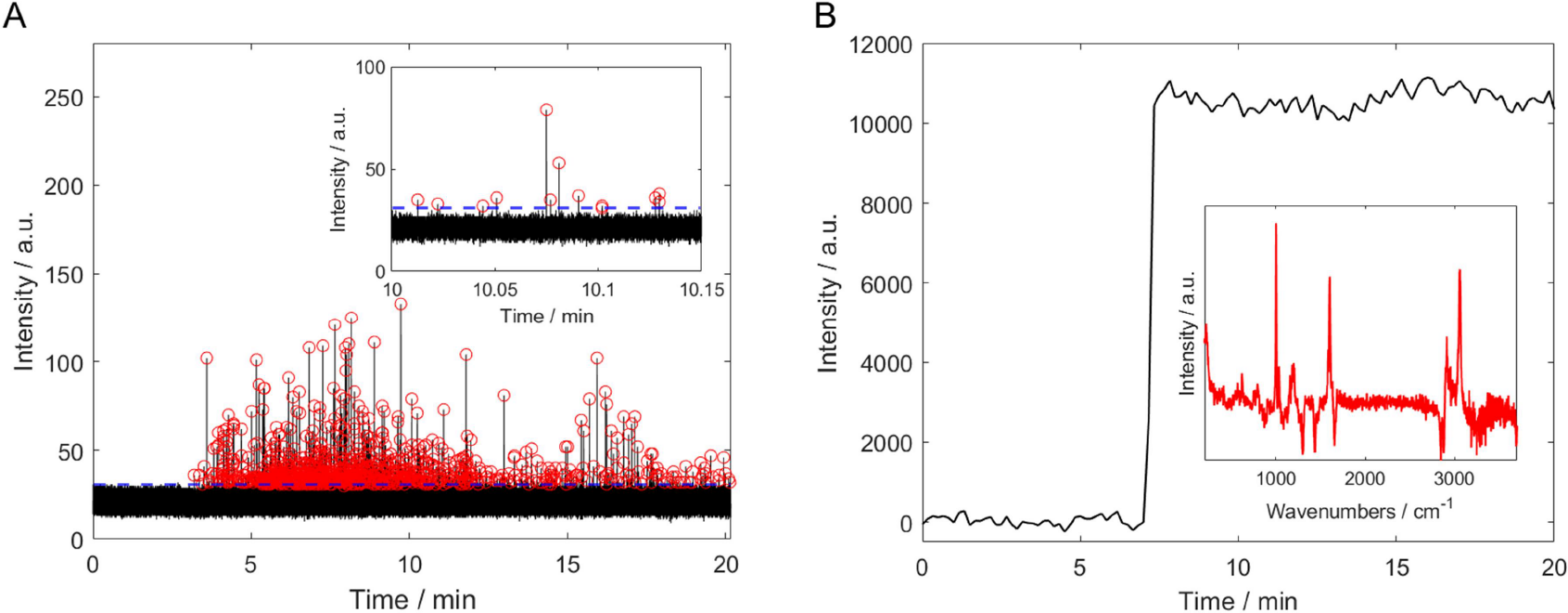
Detection of nanoplastic particle(s) in a flow-cell: Comparison of SRS (A, at 3049 cm^–1^) and spontaneous Raman (B, at 1000 cm^–1^) data for PS*X*, polydisperse PS particles with an average diameter of 250 nm. (A) Each red circle describes an event (nanoplastic particle detection represented by a peak). The dashed blue line represents the threshold which is the minimum for the peak heights to be counted. In the Supporting Information, a detailed description on how the threshold was determined is given (see also Table S2). The inset shows individual peaks of this measurement on an expanded time scale. (B) While Raman spectra in the range of 100–3785 cm^–1^ were recorded at each time point (10 s), only the intensity of the PS band at 1000 cm^–1^ is shown. One full Raman spectrum (background subtracted) can be seen in the inset of Figure 1B.

To illustrate the differences between the SRS-based setup and the previously used setup with spontaneous Raman, two measurement sets recorded for the same sample (PS*X*) are compared in Figure 1. Whereas the data acquired by spontaneous Raman show a continuously high signal when several particles are trapped and detected (see Figure 1B, starting from about *t* = 7 min), the SRS data of Figure 1A consist of individual peaks. This can be explained by the much shorter integration time of 60.5 μs for SRS, compared to 10 s for spontaneous Raman, as well as by the different degrees of OT. In the case of SRS (where little trapping was observed), it was not possible to measure close to the bottom of the flow cell where the particles would have experienced much stronger 2D-OT. To avoid high background signals, the focus position was moved 30 μm above the channel bottom. As described by Gillibert et al., this trapping in three dimensions is much weaker and less suitable for the analysis of NPLs < 500 nm with spontaneous Raman.^29^ However, due to the greater sensitivity and much better time resolution of SRS, it is possible to observe a brief, transient signal for each NPL particle passing through the focal volume. In principle, SRS-like signals could also be due to artifacts because of very strong scattering or absorption (e.g., soot particles). To verify that the observed signals are indeed due to SRS, control experiments were regularly carried out by deliberately tuning the wavenumber difference off-resonance (e.g., 3200 cm^–1^ for PS). Any artifacts would still be observed, but the true SRS signals due to PS beads are expected to disappear, as was indeed the case (see Figure S3 in the Supporting Information).

**Figure 2:**
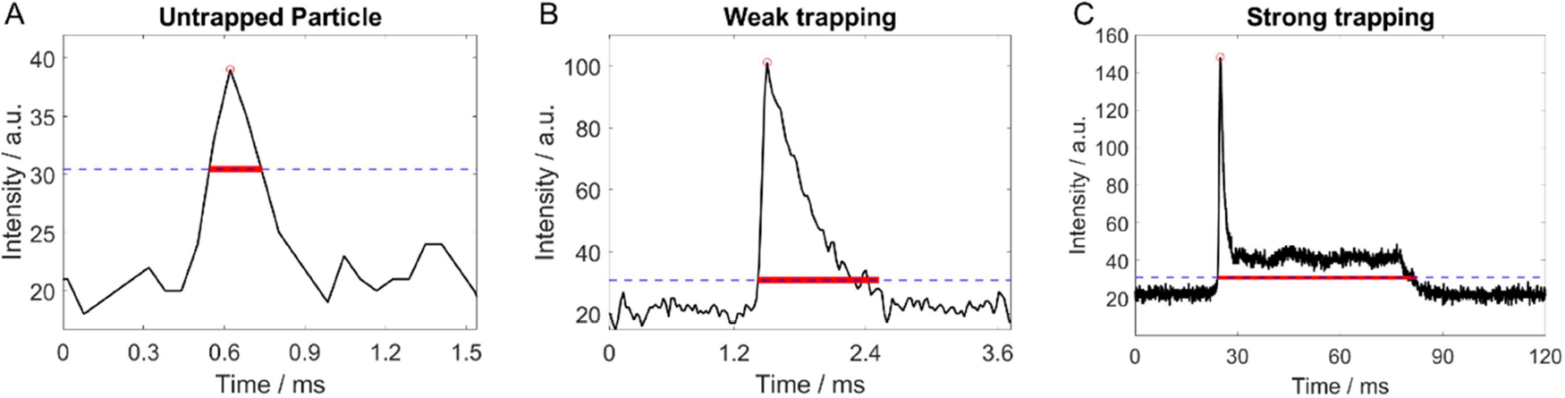
SRS data showing various degrees of OT: In the left graph A, the particle just crosses the SRS focal spot without being influenced by the optical forces; the central (B) and right graph (C) show the case of weaker or stronger trapping, respectively. The strong trapping was mainly observed for larger particles (PS1000), while the weaker and nontrapped particles are illustrated for 250 nm PS*X* in this case. The horizontal dashed blue line indicates the SRS detection threshold, while the red line indicates the peak width, as discussed further in the Supporting Information. NB: note the different time scales of the three subgraphs, each relative to an arbitrary starting point.

A closer investigation of the individual peaks in typical SRS time traces reveals different peak shapes (see Figure 2). This can be attributed to various degrees of OT. A symmetrical Gaussian-like shape (Figure 2A) indicates that the particle was not influenced by the optical forces but only passed through the focal volume. This shows the strength of SRS for the analysis of nanometer-sized individual particles, as OT is not needed for detection. The peak width and intensity provide information on the particle diameter, as will be discussed below and in the Supporting Information. Other peak shapes (see Figure 2B, C) indicate interactions with the optical forces. Figure 2B shows the response in the case of weak trapping; after about a millisecond, the particle is lost and the SRS signal returns to baseline. For strongly trapped particles (Figure 2C), it was observed numerous times that the peak showed an initial spike followed by a relatively constant signal at lower intensity. Since the scattering force is stronger than the gradient force at the focal spot of the laser beam, particles are trapped at an equilibrium position below the focal spot.^31^ For confocal, nonlinear microscopy, this results in a decreased signal intensity (see Figure S4 in the Supporting Information for an explanation). Trapping also results in a significantly higher peak width (from hundreds of μs for untrapped particles to tens of ms for strongly trapped particles). Furthermore, the peak shapes indicate that particles are detected individually.

**Figure 3:**
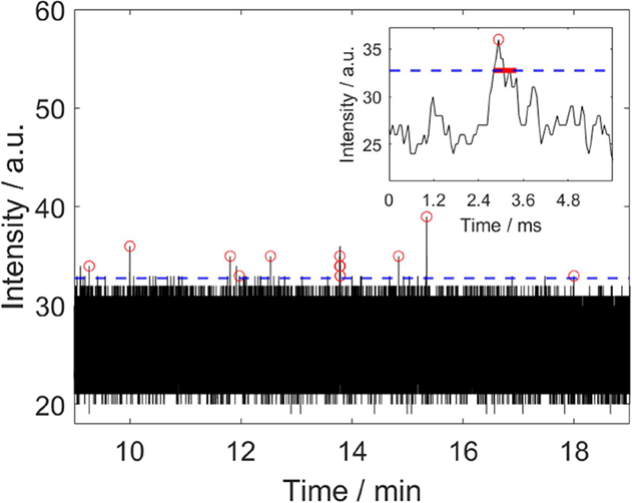
SRS detection of 100 nm PMMA particles in transmission at 2955 cm^–1^: Each red circle describes an event (nanoplastic particle detection represented by a peak). The inset shows an individual peak on an expanded time scale. The dashed blue line represents the threshold which is the minimum for the peak heights to be counted.

With this setup, it was possible to measure PS particles in a range from 230 to 5000 nm in epi-detection. The used reference/test materials consisted not only of monodisperse materials but also polydisperse or irregular-shaped particles to test the applicability of this technique for more realistic samples. However, it is difficult to draw conclusions from the statistics of the latter. Furthermore, the detection of PE and PMMA particles in that size range was also successful. However, in order to detect 100 nm particles in flow, the use of higher laser powers was necessary (Figure 3). For that reason, the gold-plated flow cell was changed to transparent PC for detection in transmission, which meant that twice the laser power (66 mW for Stokes and 34 mW for pump) could be applied at the sample. In total, the whole range of tested particles could be detected.

### Quantitative Analysis of NPLs

**Figure 4:**
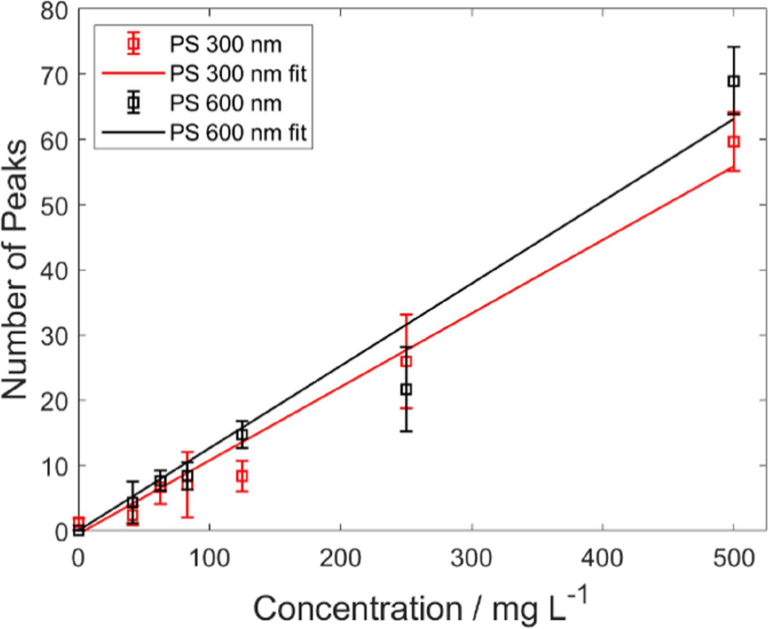
Calibration curves (detected number of particles vs mass-based concentration) for PS300 (red) and PS600 (black) using a linear weighted fit (solid lines). Both samples were measured at 3049 cm^–1^ using the same settings in epi-mode.

Since the SRS setup with the PC flow cell enables us to perform NPLs analysis at the single particle level, the number of peaks per measurement can be used to estimate the concentration (particles per volume) of the injected sample. As an example, the resulting linear calibration curves for PS300 and PS600 are shown in Figure 4. The estimation of concentrations is only reliable for higher concentrations due to the relatively high standard deviations for low particle counts. Therefore, this approach is only suitable for NPL quantification at high particle concentrations. One reason for the low sensitivity compared to proper nanoparticle quantification methods, such as NTA or CLS, is the ratio of focal and flow cell cross sections. From this value, the ratio of detectable vs injected particles can be estimated to be around 7.4 × 10^–6^ (292 detected PS600 particles for an injected concentration of 500 mg L^–1^). In other words, in our current setup, most particles will miss the very small SRS focal volume and remain undetected. Therefore, to increase the sensitivity, this ratio must be improved in the future by an optimized flow cell design. The calculations are presented in the Supporting Information. Furthermore, it has to be noted that the number of detected particles is even slightly lower for PS300 compared to the larger 600 nm beads, even though an 8-fold larger number of particles is injected at the same mass concentration due to the two times smaller particle diameter. Thus, we would expect 8 times more events per measurement. This deviation can be caused by the lower probability of smaller particles being detected because of the smaller effective focal cross section (see Supporting Information). This decreases the ratio of number of expected detections from 8 times to 5.91 times for these samples. Furthermore, the strength of optical forces acting on smaller particles is lower,^41^ and therefore, particles are less likely to be pulled toward the center of the focal volume where a suitable signal (above the threshold) can be obtained. Additionally, the mean signal intensity of smaller particles is closer to the threshold, which might lead to an underestimation of smaller particles if many of them have a signal below the threshold. More details on the signal intensity dependency on particle size can be found below. Therefore, individual calibrations for different particle size ranges (and presumably also for each polymer type) have to be performed. The width of the size range where one calibration is valid depends on the error that one is willing to accept, similar to other techniques, including NTA.

### Size Estimation Using SRS Data

**Figure 5:**
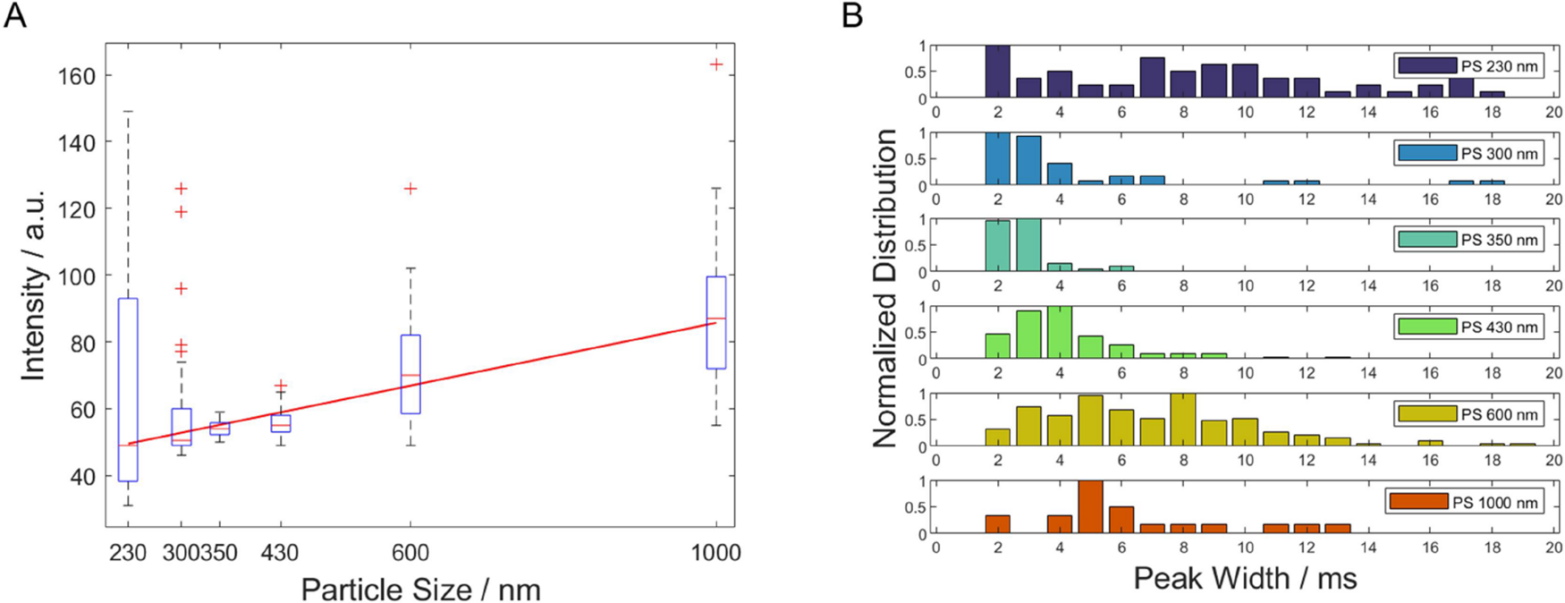
Size estimation using signal intensity (A) and peak width of untrapped particles (B): (A) Solid line is calculated using a linear weighted fit to show the trend of peak intensity with increasing particle size. (B) The normalized distributions of the peak widths are presented for each sample. The experimental peak widths were derived from the crossing points of the peak with the threshold, as illustrated in Figure 2A. Note that for the 230 nm particles, agglomeration was observed. Theoretical peak width calculations are shown in the Supporting Information.

Besides material identification and particle quantification, SRS data can also provide information on particle size if the measurement is performed in flow. As can be seen in Figure 5, there is a dependency between bead size and mean peak intensity/width. However, for a proper evaluation, only signals of untrapped beads can be used because OT results in variations in these parameters as described above and depends on multiple sample properties (e.g., size, shape, and refractive index). For this study, peaks were classified using their shape according to different degrees of trapping (see above and Figure 2 for reference). This was implemented in the MATLAB script shown in the Supporting Information. From this, it was also observed that larger particles are more likely to be influenced by optical forces. For 1000 nm particles, 82% ± 1.8% of the detected particles showed this influence, while only 37% ± 3.5% of detected 300 nm particles showed signals with a corresponding shape. The peak intensity and width also depend on the location where the bead passes the focal volume. If the overlap of the bead and the focal volume is low, the resulting peak will be narrower and less intense compared to a signal from a bead passing through the center of the focal volume. Since this location is unknown for a given signal/particle, a sufficiently large number of particles is needed for this size estimation (Table S1). Figure 5B shows that even for monodisperse samples, relatively broad distributions of temporal peak widths were obtained. Further theoretical considerations regarding the distribution of trajectories how a particle passes the focal volume, and its influence on the peaks, can be found in the Supporting Information. This variance in the peak height and width per particle size stresses the need for a flow cell design adapted for SRS measurements (e.g., hydrodynamic focusing flow cell) that would lead to better statistics. Furthermore, theoretical peak width values were calculated (see Supporting Information), as shown in Figure S5. The crossing points of the peak with the threshold were used to derive the experimental peak widths. For most samples, the experimentally determined values matched the calculated peak widths well. The size resolution of the setup depends on the pixel dwell time (1 pixel in time corresponds with 150 nm forward displacement at a flow rate of 0.1 mL min^–1^ or a velocity of 2.5 mm s^–1^), and therefore, a faster and less noisy setup would increase the resolution further. A correlation of SRS peak width and intensity for different particle sizes is shown in Figure S6 in the Supporting Information.

## Conclusions

In this work, SRS was demonstrated to be a powerful technique for the analysis of NPLs in flow. It was shown to be suitable for the detection of PE, PMMA, and PS in the size range of 100–5000 nm. For each polymer type, an optimal SRS wavenumber was used to add chemical selectivity. Furthermore, this technique can be used to derive additional parameters besides chemical information, i.e., concentration and particle size at the individual particle level, which is not straightforward with spontaneous Raman. Whereas in the case of spontaneous Raman optical trapping is necessary due to its inherently lower sensitivity, SRS has no need for OT and can even be used to distinguish different degrees of optical trapping. Furthermore, the much shorter measurement time per particle enables the detection of more individual particles, which would result in better statistics. In addition to the quantitative analysis of NPLs with SRS by means of particle counting, the mean peak width and intensity can be correlated to the particle size, statistically providing a rough size estimation. Furthermore, these results indicate that hyphenation with FFF is possible. It should be noted that with the current setup, the statistical probability of a particle flowing through the SRS focal volume is very low. By using a flow cell with a smaller channel cross section or hydrodynamic focusing,^42^ the sensitivity toward lower particle concentrations might be improved. For real samples, the major limitation is that with picosecond SRS, only a single vibration can be observed at a time and thus unambiguous chemical identification of individual particles is not possible in a flow-based setup. However, this can be overcome by the use of broadband SRS which could be applied to monitor multiple wavelengths at the same time.^43,44^ Overall, online-SRS detection can provide an extensive data set within one measurement and thus can be used to estimate multiple properties of the NPLs in the sample.

## Supporting Information Available

Additional schematic representations of the experimental details, additional experimental data, including off-resonance measurements, theoretical calculations, and the MATLAB code used for data evaluation (PDF)
