# Supplementary material for: Multi-Parameter Analysis of Nanoplastics in Flow: Taking Advantage of High Sensitivity and Time Resolution Enabled by Stimulated Raman Scattering
Source: Anal Chem. 2024 May 21;96(22):8949–55. doi: 10.1021/acs.analchem.3c05881 (PMC11154663; doi:10.1021/acs.analchem.3c05881)
Supplement: Supplementary file 1 — ac3c05881_si_001.pdf [file ac3c05881_si_001.pdf]

Supporting Information

with

Multi-Parameter Analysis of Nanoplastics in Flow: Taking Advantage of High Sensitivity and Time Resolution Enabled by Stimulated Raman Scattering

Maximilian J. Huber[1], Liron Zada[2], Natalia P. Ivleva[1,*], Freek Ariese[2,*]

Author information

1) Maximilian J. Huber (ORCID 0000-0003-3734-2103), Natalia P. Ivleva (ORCID 0000-0002-7685-5166): Chair of Analytical Chemistry and Water Chemistry, Institute of Water Chemistry, Technical University of Munich, Lichtenbergstr. 4, 85748, Garching, Germany
2) Liron Zada (ORCID 0000-0002-8419-1945), Freek Ariese (ORCID 0000-0002-8756-7223): LaserLaB Amsterdam, Department of Physics and Astronomy, Vrije Universiteit Amsterdam, 1081 HV Amsterdam, The Netherlands

Corresponding authors:
Natalia P. Ivleva (natalia.ivleva@tum.de) and Freek Ariese (f.ariese@vu.nl)

Table of contents

Figure S1: Schematic of the flow cell with a gold-plated steel base S2
Figure S2: Schematic of the SRS setup S2
Figure S3: Illustration of a typical control experiment with 300-nm PS beads, with the wavenumber difference mistuned to 3200 cm^{-1} S3
Figure S4: Schematic representation of OT of a single particle with the resulting signal intensity S3
Figure S5: Size estimation using SRS peak width S4
Figure S6: Correlation of peak width and intensity for different particle sizes S4
Estimator for goodness of particle size determination S5
Ratio of detected particles vs injected particles S5
Theoretical peak width S6
SRS data evaluation S6

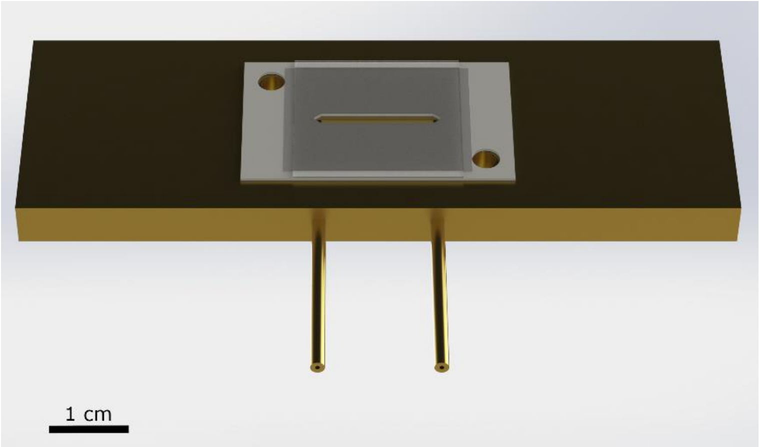

Figure S1: Schematic of the flow cell with a gold-plated steel base. The 3D-rendering was created using SOLIDWORKS 2021. The flow cell used for transmission measurements consisted of a polycarbonate base with a similar construction and the same arrangement of adhesive tape, spacer and glass cover slip. For both cells, the channel had a width of 1.5 mm and a thickness of 450 µm.

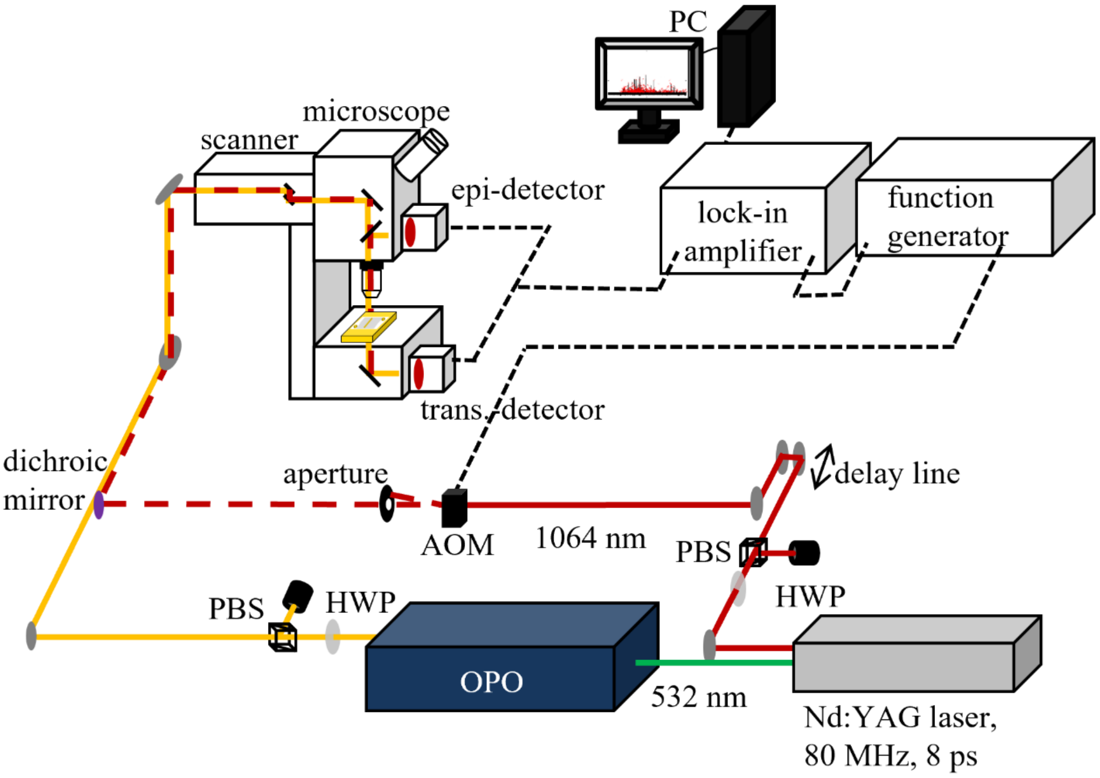

Figure S2: Schematic of the SRS setup: HWP: half-wave plate; PBS: polarizing beam splitter; AOM: acousto-optic modulator. Further information on the setup can be found in[1,2].

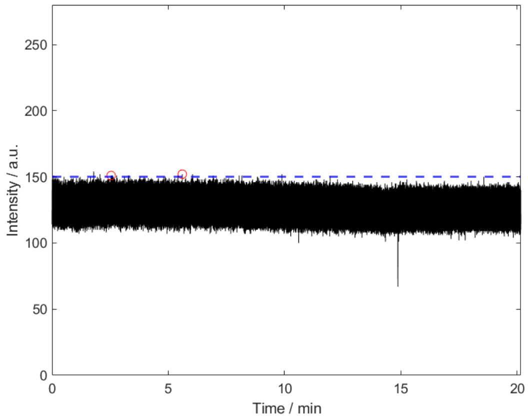

Figure S3: Illustration of a typical control experiment with 300-nm PS beads, with the wavenumber difference mistuned to 3200 cm^{-1}: As expected, the positive PS-related peaks disappeared, apart from two false positives, confirming that the signals (e.g. Figure 1 in the main text) were indeed due to SRS and not to scattering/absorption artefacts that are much less wavenumber-specific. Note that the background is higher here, due to an increased signal from the water O-H band. The single negative peak at about 14.9 min was probably due to a large dust particle, resulting in a temporary decrease of the water background signal.

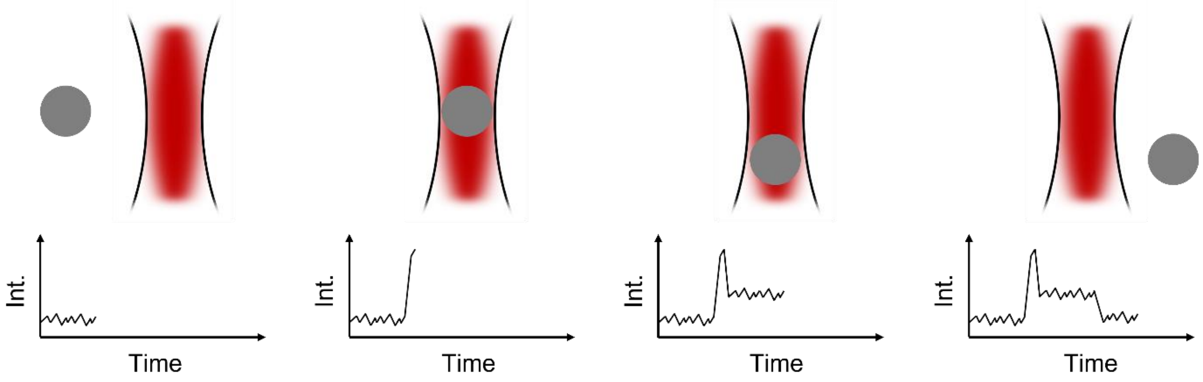

Figure S4: Schematic representation of OT of a single particle with the resulting signal intensity: First, the particle is not yet in the focal volume of the lasers, resulting in background signal intensity level. In the next graphic the particle has entered the focal volume at the center resulting in the highest possible signal intensity. Due to the dominating scattering force at the center (in our setup the laser irradiation occurs from the top), the particle is pushed down to an equilibrium position where scattering and gradient force are equal. This process reduces the acquired signal intensity. Due to heating, the trap destabilizes after some time and the particle is released with the signal returning to background levels.

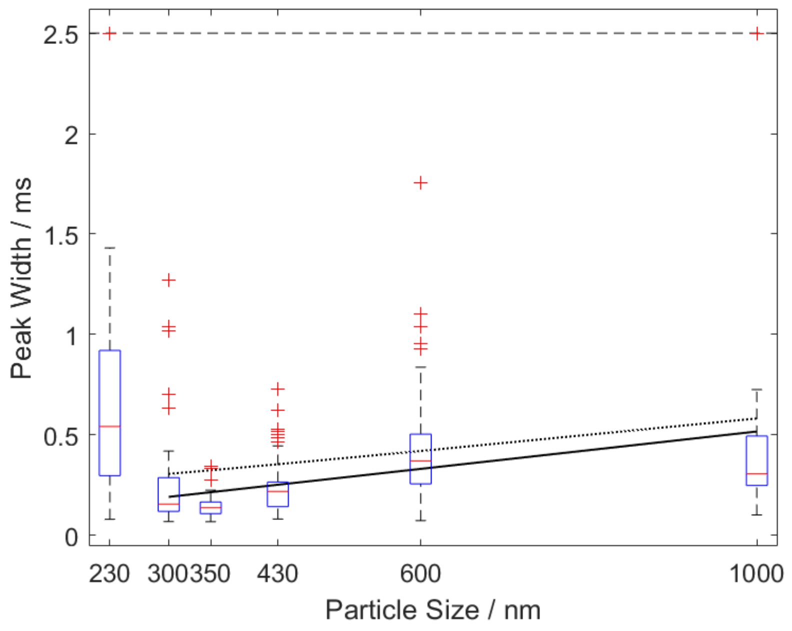

Figure S5: Size estimation using SRS peak width: The solid lines are calculated using a linear weighted fit to show the trend of peak width with increasing particle size (230 nm excluded due to agglomeration). The dotted line represents the theoretical peak width (for the calculations see below). The experimental peak widths were derived from the crossing points of the peak with the threshold as illustrated in figure 2A of the main text.

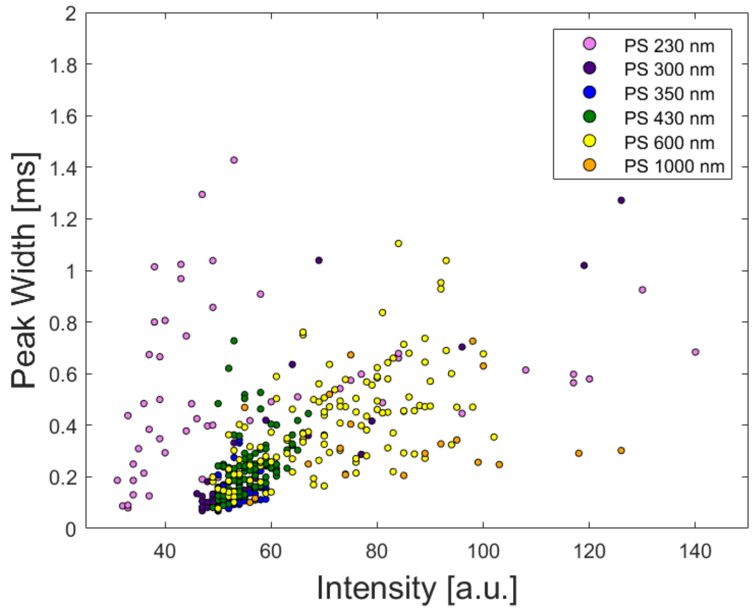

Figure S6: Correlation of peak width and intensity for different particle sizes.

Estimator for goodness of particle size determination

To get an idea if sufficient particles have been detected for reliable particle size estimation within the instrumental limits, an estimator E_i (eq. 1) can be used. For this, we introduce the number of detected particles n_i of a sample i in addition with the 10th and 90th percentile of the peak widths of these particles, $pw_{10,i}$ and $pw_{90,i}$.

$$E_i = n_i^{-2} \cdot \frac{pw_{90,i} - pw_{10,i}}{pw_{10,i}} \tag{1}$$

The resulting values are summarized in Table S1. Lower values suggest that sufficient particles have been measured, while the threshold must be chosen individually. For polydisperse samples, more particles have to be measured to get a good representation of the sample and low estimator values.

Table S1: Resulting estimator values for different samples.

Particle size	PS230	PS300	PS350	PS430	PS600	PS1000
Estimator E_i	0.85%	0.48%	0.09%	0.02%	0.02%	1.30%

Ratio of detected particles vs injected particles

To estimate the number of theoretically detectable particles $n_{det,theo}$ (eq. 2) we assume a uniform distribution of particles over the channel cross section. This assumption is mainly based on the neglectable influence of gravity under the given flow conditions and time scales on nanoplastics.[3] Thus, it depends on the number of injected particles n_{inj} and the ratio of the focal and channel cross sections: $\frac{A_{foc}}{A_{ch}}$.

$$n_{det,theo} = n_{inj} \cdot \frac{A_{foc}}{A_{ch}} = \frac{V_{inj} \cdot c_{inj}}{\frac{4}{3}\pi \cdot r_P^3 \cdot \rho_P} \cdot \frac{A_{foc}}{A_{ch}} \tag{2}$$

The number of injected particles can be calculated from the injection volume V_{inj}, the mass concentration c_{inj} and the mass of one, spherical particle $m_P = \frac{4}{3}\pi r_P^3 \cdot \rho_P$, with r_P and ρ_P as the particle radius and density, respectively.

$$A_{foc} = \pi \cdot (\beta \cdot h_{foc} + \alpha \cdot r_P) \cdot (\beta \cdot w_{foc} + \alpha \cdot r_P) \tag{3}$$

The effective focal cross section A_{foc} is calculated as an ellipse (eq. 3) with $h_{foc} = 2\ \mu m$ and $w_{foc} = 0.6\ \mu m$. The particle radius r_P and the dimensions of the focal spot are corrected by the corresponding factors α and β. This is done because a significant overlap between the particle and the focal volume is needed to generate a signal. Since the beam shape is Gaussian, we used the 1σ-criterion to allow for a significant overlap using $\beta = 0.6827$. Accordingly, it was chosen that 68.27% of the particle volume must be within the focal volume. Thus $\alpha = \sqrt[3]{0.6827} = 0.8805$ was used to correct the particle diameter. Thus, for 500 mg L^{-1} PS600 and PS300 samples $n_{det,theo}$ would be around 292 and 1547 particles per injection, respectively. The applied SRS detection threshold (see also below) means of course that the number of actually counted particles will be lower than the nominal number of particles traveling through the focal volume. Non-uniformity in the particle distribution and a non-ideal overlap of the two laser beams might also lead to a lower number of detected particles.

Theoretical peak width

The theoretical peak width w_S (= duration for an untrapped particle, eq. 4) can be estimated from the flow rate $\dot{V}$ and the particle and focal spot diameter calculated above. At the tested flow conditions (2.5 mm s^{-1}) and particle sizes (100 - 5000 nm) no distortion due to diffusive motion is expected. This was for example shown by Matsuura *et al.* for an even lower flow velocity of 100 µm s^{-1} and with smaller particles (30 nm and 100 nm).[4] The volumetric flow rate must be converted to the flow velocity using the channel cross section A_{ch}.

$$w_S = \frac{2r_P \cdot \alpha + w_{foc} \cdot \beta}{\frac{\dot{V}}{A_{ch}}} \tag{4}$$

SRS data evaluation

The code below was used to evaluate the SRS data relative to a threshold value. For this, STD = 4.5 and CUT = 3.0 were chosen. Selecting a smaller value for CUT resulted in overestimated peak widths since in some cases the end of the peak would be set to the end of the measurement. For STD we assumed the distribution of the noise to be Gaussian. This was confirmed by a QQ-plot using MATLAB, which showed high linearity. Based on the threshold $\theta = \mu + STD \cdot \sigma$, with µ and σ being the mean and the standard deviation of the noise and

STD the number (multiplier) of σ, we can calculate the probability of two adjacent noise data points being erroneously picked as a signal (false positive). A peak with a width less than two data points is not counted since the autocorrelation function in MATLAB showed that two adjacent data points depend on each other. Thus, the number of false positives is mainly determined by the probability of two adjacent data points being above the threshold (Table S2). This might be caused by a slow response time in the electronics. Using this information, we can calculate the number of false positives for one 20-min measurement of $2 \cdot 10^7$ data points, assuming independent data points:

Table S2: Calculation of false positives for various values of the threshold in units of standard deviation.

Number of standard deviations (STD)	Probability P for one data point to be noise[1]	Number of false positive signals $(1-P)^2 \cdot 2 \cdot 10^7$
3.0	0.9973002	146
3.5	0.9995347	4.5
4.0	0.9999367	0.1
4.5	0.9999932	0.001
5.0	0.9999994	0.000005

[1] calculated from the cumulative distribution function of a normal distribution

Since it was shown that the data points are not independent and to account for deviations from a perfect Gaussian distribution, we selected STD to be 4.5 instead of 4.0.

Code used for the general data evaluation for time series SRS data on nanoplastics in flow:

```
function SRS_eval(tif_file_name, z_STD, z_CUT)
    arguments
        tif_file_name (1,:) char
        z_STD (1,:) double
        z_CUT (1,:) double
    end

    %GOAL: Import data from Zeiss microscope stored as .tif file
    A=double(imread(tif_file_name)); %read tiff file
    B=transpose(A(:,:,1)); %preapare to read in the correct direction
    Array=B(:); %read matrix into array
```

```
    %SECTION STATISTICS & PEAK PICKING

    %GOAL: Find peaks based on mean and STD of noise
    b=length(Array); %number of data points
    X=(transpose(double(1):double(b))*0.0000605); %generate x values in seconds
    X_min=X/60; %x values in minutes
    M_n=mean(Array(1000000:4000000,1)); %mean intensity of noise, depending on measurement
conditions data range may have to be adapeted
    S_n=std(Array(1000000:4000000,1)); %std of noise, depending on measurement conditions data
range may have to be adapeted
    threshold=M_n+z_STD*S_n; %threshold of noise for peak finding
    [pks,locs,w,p] = findpeaks(Array,'MinPeakHeight',threshold); %find peaks above threshold,
pks = peak max. intensity, locs = peak position, w = peak width, p = peak prominence
    test = ones(1); % preallocation of variable
    %delete peaks that are counted multiple times
    for k = 1:(length(pks)-1) % loop over all peaks except last
        m = locs(k):locs(k+1); % m is region between peaks
        n = m(2):m(end-1); % n is region between peaks excluding start/end point
        test(k) = any((Array(n) < M_n)); %test if any of the measurement points of a peak are
below noise average; if this is not the case, the same peak was counted multiple times
    end
    t_corr=~[1 test]'; % add one to keep the first peak; this shows which peaks to delete from
peaks list
    if ~isempty(pks) %delete peaks from pks, locs, w & p
        pks(t_corr) = [];
        locs(t_corr) = [];
        w(t_corr) = [];
        p(t_corr) = [];
    end

    locs_corr=(locs*0.0000605)/60; %converting peak location to minutes
    locs_corr2=locs_corr./60.5.*60.*1000000; % converting from min to 1 us

    figure % create figure for all peaks found according to threshold
    hold on
    plot(X_min,Array,'k') %plot data
    yline(threshold, 'b', 'LineWidth', 1.5); %plot threshold
    plot(locs_corr,pks,'or'); %plot found peaks
    set(gca,'box','on')
    xlim([0 X_min(end)]);
    ylim([0 280]);
    xlabel('Time / min');
    ylabel('Intensity / a.u.');
    title(tif_file_name);
    hold off


    %SECTION CALCULATION OF PEAK WIDTH

    %GOAL: Convert data to integer to calculate the peak width based on the intersection with
the threshold
    cutoff=M_n+z_CUT*S_n; %define cutoff for peak width determiation to circumvent the
following problem: peak is cut to early due to noise
    X_w=uint64(X*1000000); % converting x values to 1 us & integer
    locs_corr2_w=uint64(locs_corr2); %converting peak position in 1 us to integer

    %GOAL: Calculate peak width
```

```
    for n = 1:length(locs)
        % find left intercept of each peak
        idx = locs(n);
        while Array(idx) > threshold
            idx = idx -1;
        end
        if threshold-Array(idx) > 1
            intercept_L(n)=idx+((threshold-Array(idx))/(Array(idx+1)-Array(idx)));
%interpolate to find right intecept of peak
        else
            intercept_L(n)=idx; %if intercept is at threshold no interpolation is needed
        end

        % find right intercept of each peak
        idx = locs(n);
        while Array(idx) > threshold || Array(idx+1) > cutoff || Array(idx+2) > cutoff ||
Array(idx+3) > cutoff || Array(idx+4) > cutoff % account for trapping peaks (might otherwise
be cut off to early)
            idx = idx +1;
        end
        if Array(idx)-Array(idx-1) == 0
            intercept_R(n)=idx;
        elseif threshold-Array(idx) > 1
            intercept_R(n)=idx-1+((threshold-Array(idx-1))/(Array(idx)-Array(idx-1)));
%interpolate to find right intecept of peak
        else
            intercept_R(n)=idx; %if intercept is at threshold no interpolation is needed
        end
    end
    bnds=[double((intercept_L)') double((intercept_R)')]; %get starting/end points of peaks at
height of threshold in pixel
    bnds_corr=bnds.*60.5./10^6./60; %convert starting/end points of peaks to minutes
    bnds_corr2=uint64(bnds); %convert data for area calculation
    pks_width=bnds(:,2)-bnds(:,1); %calculate peak width from starting/end points
    rows2delete = (pks_width < 0.1); %delete all peaks that are part of the noise (<0.1px)
    pks_width(rows2delete) = [];
    pks(rows2delete) = [];
    locs(rows2delete) = [];
    w(rows2delete) = [];
    p(rows2delete) = [];
    locs_corr(rows2delete) = [];
    locs_corr2(rows2delete) = [];
    locs_corr2_w(rows2delete) = [];
    bnds=bnds(~rows2delete,:);
    bnds_corr=bnds_corr(~rows2delete,:);
    bnds_corr2=bnds_corr2(~rows2delete,:);

    %GOAL: correct the peak position (x,y) to position of maximum intensity
    %Note: findpeaks function finds not necessarily the peak maximum (but local maxima)
    for k = 1:length(pks) %find the x-value of maximum intensity for each peak
        m = bnds_corr2(k,1):bnds_corr2(k,2); % m is region between peaks
        [M,I] = max(Array(m),[],'all');
        pos_corr(1,k)=I; % realtive position of maximum intensity
        pks(k) = M; %maximum intensity
        locs(k) = m(1)+I-1; % absolute position of maximum intensity
    end

    %GOAL: Calculate peak width again with corrected peak positions and delete all peaks that
are part of noise
```

```
    %Note: If only this peak width calculation was used, the peak position correction would be
very CPU intensive due to a high number of noise peaks
    for n = 1:length(locs)
        % find left intercept of each peak
        idx = locs(n);
        while Array(idx) > threshold
            idx = idx -1;
        end
        if threshold-Array(idx) > 1
            intercept_L(n)=idx+((threshold-Array(idx))/(Array(idx+1)-Array(idx)));
%interpolate to find right intecept of peak
        else
            intercept_L(n)=idx; %if intercept is at threshold no interpolation is needed
        end

        % find right intercept of each peak
        idx = locs(n);
        while Array(idx) > threshold || Array(idx+1) > cutoff || Array(idx+2) > cutoff ||
Array(idx+3) > cutoff || Array(idx+4) > cutoff % account for trapping peaks (might otherwise
be cut off to early)
            idx = idx +1;
        end
        if Array(idx)-Array(idx-1) == 0
            intercept_R(n)=idx;
        elseif threshold-Array(idx) > 1
            intercept_R(n)=idx-1+((threshold-Array(idx-1))/(Array(idx)-Array(idx-1)));
%interpolate to find right intecept of peak
        else
            intercept_R(n)=idx; %if intercept is at threshold no interpolation is needed
        end
    end
    bnds=[double((intercept_L)') double((intercept_R)')]; %get starting/end points of peaks at
height of threshold in pixel
    bnds_corr=bnds.*60.5./10^6./60; %convert starting/end points of peaks to minutes
    bnds_corr2=uint64(bnds); %convert data for area calculation
    pks_width=bnds(:,2)-bnds(:,1); %calculate peak width from starting/end points

    test_peak_1 = (Array(locs+1) > threshold); % check if pixels adjacent to peak position are
above threshold
    test_peak_2 = (Array(locs-1) > threshold);% check if pixels adjacent to peak position are
above threshold
    rows2del = ~any([test_peak_1 test_peak_2],2); %delete all peaks that are part of the noise
(<2px)
    pks(rows2del) = [];
    locs(rows2del) = [];
    w(rows2del) = [];
    p(rows2del) = [];
    locs_corr(rows2del) = [];
    locs_corr2(rows2del) = [];
    locs_corr2_w(rows2del) = [];
    bnds=bnds(~rows2del,:);
    bnds_corr=bnds_corr(~rows2del,:);
    bnds_corr2=bnds_corr2(~rows2del,:);
    pks_width=bnds(:,2)-bnds(:,1);

    pks_width_min=pks_width.*60.5./10^6./60; %convert width in pixels to width in minutes
    M_pw = mean(pks_width_min); %calculate the mean peak width
    S_pw = std(pks_width_min); %calculate the standard deviation of the peak width
```

```
    %GOAL: Figure including peak width and threshold as line
    figure %plot figure with peaks (excluding noise peaks)
    hold on
    plot(X_min,Array,'k') %plot data
    yline(threshold, 'b', 'LineWidth', 1.5); %plot threshold
    plot(locs_corr,pks,'or'); %plot found peaks
    for n=1:length(pks)
        hL(n)=line(bnds_corr(n,:),[threshold threshold],'color','r', 'LineWidth', 3); %loop to
plot peak width for every peak
    end
    set(gca,'box','on')
    xlim([0 X_min(end)]);
    ylim([0 280]);
    xlabel('Time / min');
    ylabel('Intensity / a.u.');
    title(tif_file_name);
    hold off
    savefig([tif_file_name,'_G1.fig']); %save figure as MATLAB image
    ax = gca; %export figure as eps and png
    exportgraphics(ax, [tif_file_name,'_G1.eps']);
    exportgraphics(ax, [tif_file_name,'_G1.png']);


    %SECTION CALCULATE AREA & AVERAGE INTENSITY

    %GOAL: Calculate peak area using peak width and correction for noise, loop integration
over all peaks to get peak area
    area=zeros([1,length(pks)]); %preallocating variable
    for n=1:length(pks)
        pks_int_x=double(transpose(X_w(bnds_corr2(n,1):bnds_corr2(n,2)))); %set up x limits
for peak integration, vary small peaks might have overestimated peak width, which leads to
slightly larger area
        pks_int_y=double(transpose(Array(bnds_corr2(n,1):bnds_corr2(n,2)))); %set up peak
intensity for integration
        I1 = trapz(pks_int_x,pks_int_y)/60000000-(M_n*pks_width_min(n)); %integrate over the
width of peaks to get area and correct for noise
        area(n)=I1; %write area to array
    end
    area_corr=transpose(area); %prepare array for export table

    %GOAL: Calculate the average peak intensity for each peak
    avg_int=area_corr./pks_width_min; %calculate average peak intensity per peak
    M_int=mean(avg_int); %calculate the average peak intensity over all peaks
    S_int=std(avg_int); %calculate the corresponding standard deviation
    int_ratio = pks./avg_int; % calculate the ratio of maximum intensity to average intensity


    %SECTION PEAK CLASSIFICATION: SHORT GAUSSIAN; EXP DECAY; TRAPPING

    %GOAL: define suitable classes for diffrernent peak shapes
    class = repmat("normal peak ", [length(pks) 1]);
    class_data=table(class);
    % slope_comp=(locs_corr(16)-bnds_corr(16,1))/(bnds_corr(16,2)-locs_corr(16))
    for i=1:length(pks)
        slope_comp=(locs_corr(i)-bnds_corr(i,1))/(bnds_corr(i,2)-locs_corr(i));
        if slope_comp>0.3
            class_data{i,"class"}="no opt. trap.";
        elseif slope_comp>0.02
            class_data{i,"class"}="weak trapping";
```

```
        else
            class_data{i,"class"}="int trapping ";
        end
    end

    events=length(pks);
    if events==0
        Mean=table(events,M_n,S_n,M_int,S_int,M_pw,S_pw); %general information on data
        writetable(Mean, [tif_file_name,'_info.txt']); %save general information on
measurement data
    else
        Mean=table(events,M_n,S_n,M_int,S_int,M_pw,S_pw); %general information on data
        pks_data=table(locs_corr,pks,avg_int,int_ratio,pks_width_min,area_corr); %information
on every peak
        pks_class_data=[pks_data class_data]; %information on every peak including
classification
        writetable(pks_class_data, [tif_file_name,'_output.txt']); %save peak specific
information as text file
        writetable(Mean, [tif_file_name,'_info.txt']); %save general information on
measurement data
    end
    clearvars;

end
```

References

(1) Zada, L.; Leslie, H. A.; Vethaak, A. D.; Tinnevelt, G. H.; Jansen, J. J.; de Boer, J. F.; Ariese, F. Fast Microplastics Identification with Stimulated Raman Scattering Microscopy. *J. Raman Spectrosc.* **2018**, *49* (7), 1136–1144. https://doi.org/10.1002/jrs.5367.

(2) Haasterecht, L.; Zada, L.; Schmidt, R. W.; Bakker, E.; Barbé, E.; Leslie, H. A.; Vethaak, A. D.; Gibbs, S.; Boer, J. F.; Niessen, F. B.; Zuijlen, P. P. M.; Groot, M. L.; Ariese, F. Label-free Stimulated Raman Scattering Imaging Reveals Silicone Breast Implant Material in Tissue. *J. Biophotonics* **2020**, *13* (5). https://doi.org/10.1002/jbio.201960197.

(3) Gigault, J.; El Hadri, H.; Nguyen, B.; Grassl, B.; Rowenczyk, L.; Tufenkji, N.; Feng, S.; Wiesner, M. Nanoplastics Are Neither Microplastics nor Engineered Nanoparticles. *Nat. Nanotechnol.* **2021**, *16* (5), 501–507. https://doi.org/10.1038/s41565-021-00886-4.

(4) Matsuura, Y.; Nakamura, A.; Kato, H. Nanoparticle Tracking Velocimetry by Observing Light Scattering from Individual Particles. *Sens. Actuators B Chem.* **2018**, *256*, 1078–1085. https://doi.org/10.1016/j.snb.2017.10.054.
